# A Magnetic Food Texture Sensor and Comparison of the Measurement Data of Chicken Nuggets

**DOI:** 10.3390/s21103310

**Published:** 2021-05-11

**Authors:** Hiroyuki Nakamoto, Yuya Nagahata, Futoshi Kobayashi

**Affiliations:** 1 Graduate School of System Informatics, Kobe University, 1-1 Rokkodai-cho, Nada-ku, Kobe 657-8501, Japan; futoshi.kobayashi@port.kobe-u.ac.jp; 2 J-Oil Mills, Inc., 8-1, Akashi-cho, Chuo-ku, Tokyo 104-0044, Japan; yuya.nagahata@j-oil.com

**Keywords:** food texture, force, vibration, magnetoresisitive, magnetic sensor

## Abstract

Food texture is one of the important quality indicators in foodstuffs, along with appearance and flavor, contributing to taste and odor. This study proposes a novel magnetic food texture sensor that corresponds to the tactile sensory capacity of the human tooth. The sensor primarily consists of a probe, linear slider, spring, and circuit board. The probe has a cylindrical shape and includes a permanent magnet. Both sides of the spring are fixed to the probe and circuit board. The linear slider enables the smooth, single-axis motion of the probe during food compression. Two magnetoresistive elements and one inductor on the circuit board measured the probe’s motion. A measurement system then translates the measurement data collected by the magnetoresistive elements into compression force by means of a calibration equation. Fundamental experiments were performed to evaluate the range, resolution, repetitive durability of force, and differences in the frequency responses. Furthermore, the sensor was used to measure seven types of chicken nuggets with different coatings. The difference between the force and vibration measurement data is revealed on the basis of the discrimination rate of the nuggets.

## 1. Introduction

Food texture is an important quality indicator in foods, alongside factors such as appearance, taste, and odor [1,2,3,4]. Szczesniak defines texture as a sensory property arising from the response of the tactile senses to physical stimuli, and is a multi-parameter attribute [5]. In order to evaluate food attributes, it is necessary to establish the physical characteristics that occurred during the compression or fracture of given foodstuffs.

In terms of the evaluation of food attributes, texture profile analysis (TPA) has been widely used for various foods. Working within the TPA framework, Szczesniak divided the mechanical characteristics of physical properties into five basic parameters, namely: hardness, cohesiveness, viscosity, springiness, and adhesiveness [6,7,8]. These parameters were determined on the basis of measurement data and force versus time, and consist of two time compression and decompression curves. Fracturability is also determined from measurement data and entails a rapid drop in force. In general, instruments used for TPA measure the force by load cell at low sampling frequencies of up to 100 Hz. Hence, instruments with only load cells are insufficient for textures such as crispness and crunchiness with fracturing. As these qualities are popular in many countries [9], a novel texture sensor design is needed in order to measure the details of fractures.

Various studies have proposed methods and devices to measure the vibrations that occur during fractures. Vibration data are suitable for the evaluation of detailed textures, especially crispness and crunchiness [10]. Chen et al. analyzed the crispness of biscuits by means of an acoustic envelope detector [11]. Meanwhile, Varela et al. revealed that the crispness of almonds had a high correlation with the number of vibrations and height of the vibration peaks [12]. Taniwaki et al. developed a measurement system equipped with a piezoelectric sensor and analyzed measurement data using the fast Fourier transformation [13]. Taniwaki et al. also assessed the correlation between mechanical and acoustic characteristics [14]. In measuring the crispness of commercial potato chips, Salvador et al. employed a load cell and microphone and measured force and sound data, which were then subjected to a principal component analysis [15]. Akimoto et al. evaluated the wet crisp texture of fruits based on vibration, which they measured using a device with a free-running probe [16]. In turn, Sakurai et al. developed a measurement system for vertical and horizontal vibrations, which comprised a swingarm device with multiple accelerometer sensors that measured the vibration without the use of electric actuators [17].

The abovementioned studies revealed the importance of vibration for evaluating food texture. However, the evaluation of texture by means of both force and vibration is not a common practice by food manufacturers. We consider there to be two key problems in this area. One is that there is no standardized evaluation method for food texture. If there is sufficient measurement data, artificial intelligence-utilizing techniques may solve this problem. Neural networks have been used to estimate food texture in terms of crispness and crunchiness based on force and acoustic signals [18]. The other issue is that there is no standard sensor for simultaneously measuring force and vibration. A standard sensor could constitute an integrated-type device equipped with force and vibration sensors, for instance.

In this study, we propose a novel magnetic food texture sensor that corresponds to the tactile sense of the human tooth. We developed a magnetic food texture sensor that imitated the structure of part of the tooth [19]. A urethane elastomer within the sensor was adopted as the periodontal ligament beneath the tooth. However, the elastomer had low repetitive durability. Therefore, we redesigned it to have higher durability. The new sensor primarily consists of a probe, linear slider, spring, and circuit board. The probe is cylindrical in shape and includes a permanent magnet. Both sides of the spring are fixed to the probe and circuit board. The linear slider enables the smooth, one-axis motion of the probe during food compression. Two magnetoresistive (MR) elements and one inductor on the circuit board were used to measure the probe’s motion. The usage of the MR element and inductor is based on the report that the periodontal ligament, which is under human and animal teeth, has two types of mechanoreceptors with different response characteristics [20]. The slowly adapting type has a function to produce sustained responses to static stimulation by force. The rapidly adapting type produces transient responses to the onset and offset of stimulation by vibration. The MR elements and the inductor in the texture sensor have roles of slowly adapting and rapidly adapting, respectively. With respect to sensors using a magnet, various tactile sensors were proposed [21,22,23]. They focused on the softness of robotic skin and used soft materials as a probe or surface layer. One of their advantages was a wireless structure between the deformation part and measurement part by using the magnet. The texture sensor in this study has a common advantage of the wireless structure and has a structural difference using the probe made from hard material, the linear slider, and the spring. In the following section, the structure and calibration method of the sensor are described. Fundamental experiments were performed to evaluate the sensor’s range, resolution, repetitive durability, and frequency response. Moreover, the sensor is used to measure seven types of chicken nuggets with different coatings. The difference between the force and vibration measurement data is then revealed based on the discrimination rate of these.

## 2. Materials and Methods

### 2.1. Magnetic Food Texture Sensor

#### 2.1.1. Structure

**Figure 1:**
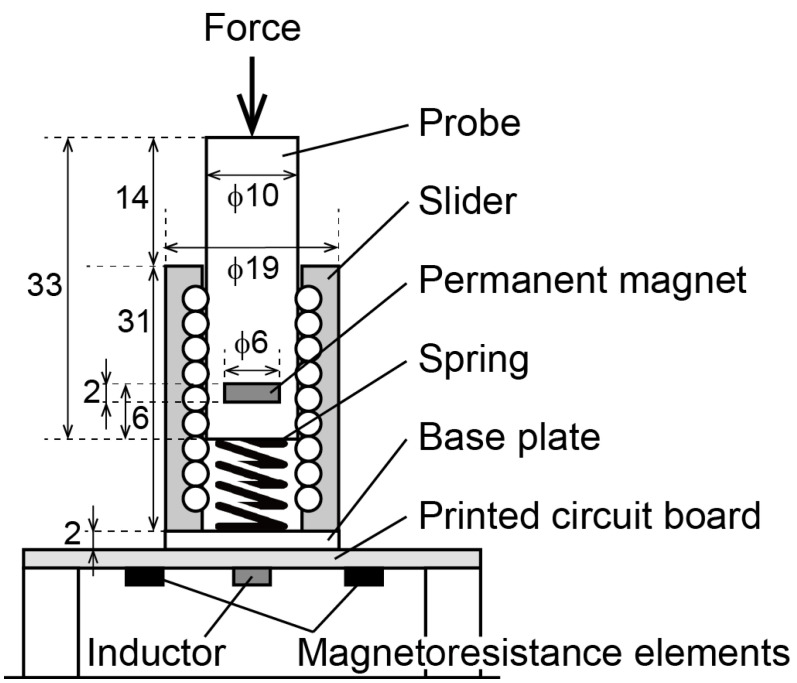
Cross-sectional view of the texture sensor.

The texture sensor developed for this study comprised four main components: A probe, linear slider, spring, and circuit board. A cross-sectional view of the probe from the perspective of the center is shown in Figure 1. The probe, which is made of acrylonitrile-butadiene-styrene resin and produced by a 3D printer, has a cylindrical shape, a diameter of 10 mm and length of 33 mm, and contains a permanent magnet. The top and bottom surfaces of the probe are flat. The weight of the probe is 2.5 g. Below the probe is an industrial spring, both ends of which are fixed to the bottom of the probe and top of the base plate. The linear slider (LM10UU, THK Co., Tokyo, Japan) has a ball bearing on the inner surface and enables smooth vertical movements of the probe, as is displayed in Figure 1. The circuit board is adhered to the base plate and an inductor (LQH43NN242K03L, Murata Manufacturing Co., Kyoto, Japan)is soldered to it below the probe, and two MR elements (AA003-02E, NVE Co., Minneapolis, MN, USA) are also soldered onto each side of the inductor. The texture sensor is fixed to a motorized slider with a vertical inversion capacity. The motorized slider compresses or breaks food at the tip of the probe. When this occurs, the strength of the magnetic field with respect to the MR elements and inductor changes as the probe displaces in the direction of the arrow shown in Figure 1. The output voltage of the MR elements is proportional to the strength of the magnetic field. The inductor generates an induced electromotive force that is proportional to the change in the magnetic flux density in accordance with Faraday’s law of electromagnetic induction. The texture sensor has a wireless structure between the probe and the sensor elements. This structure has two advantages. First, the MR elements and inductor measure the motion of the probe as the change of the magnetic field without joints and wire. Second, it is easy to clean the sensor. Most food includes moisture and oil. As this structure separates the probe from the sensor elements by the circuit board, there is no need to clean or replace the sensor elements.

#### 2.1.2. Amplifier and Quantization Circuit

**Figure 2:**
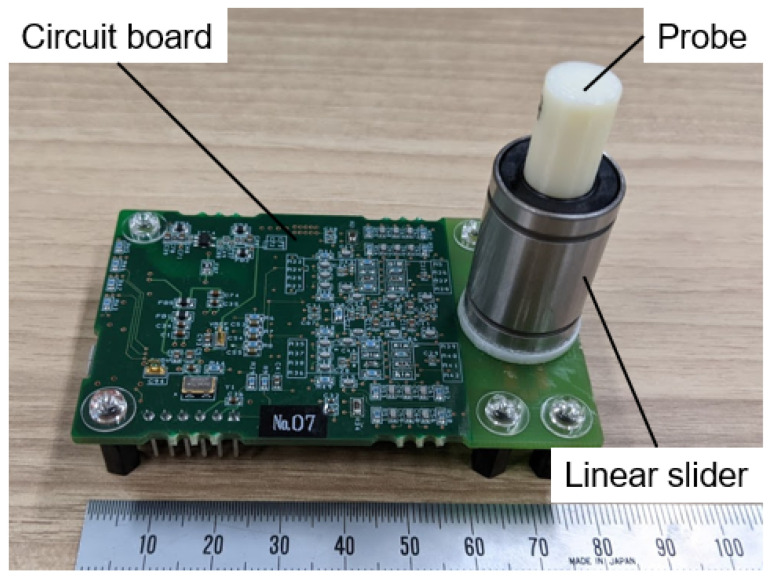
Prototype of the texture sensor. The circuit board includes an amplifier circuit and a microcomputer with AD conversion ports.

Depending on the displacement of the probe, the MR element and inductor output static and dynamic voltages, respectively, relative to the strength of the magnetic field. As the range of these voltage changes is small, the amplifier circuit amplifies the voltages of the MR elements and inductor by 59.4 times and 5940 times, respectively. Due to the usage of an inversion amplifier, the range of the amplified output of the MR element is from 3.8 to 1.0 V. Subsequently, a low-pass filter with a cutoff frequency of 4.8 kHz removes both the random noise and aliasing noise of those voltages. The noise-removed voltages are then converted into digital data by a microcomputer equipped with an AD conversion port. The sampling frequency of the measurement is 10 kHz. The measurement data is then transmitted to a desktop computer via the communication port of the microcomputer and USB cable. A prototype of this unit is shown in Figure 2. The right side of Figure 2 shows the probe and linear slider. The left side of Figure 2 displays the amplifier and quantization circuit. The circuit board integrates these components.

#### 2.1.3. Calibration and Range of Force

As the probe is supported by the spring, its displacement is proportional to the force acting on the probe’s tip. The relationship between the displacement and voltages of the MR elements is not linear. Hence, Equation (1) was determined by trial and error by adding low-order terms and a constant term based on Coulomb’s law, as a calibration equation, calculates the force *F* acting on the texture sensor’s probe:

(1) $$F=a_{0}+\frac{a_{1}}{v_{1}}+\frac{a_{2}}{v_{1}^{2}}+\frac{a_{3}}{v_{2}}+\frac{a_{4}}{v_{2}^{2}}$$

where $a_{i}\left(i=0,\ldots ,4\right)$ are coefficients determined by a calibration, and $v_{1}$ and $v_{2}$ are the amplified voltage of the MR elements. In order to acquire the calibration data, determine the compression length of the spring from the target force range, divide the length was determined, then divided into 20 equal lengths, and measure the combination of the force and the voltages of the MR elements at each compression length measured. The reference force was measured by a load cell. The parameters of the calibration equation were determined by means of the least-squares method using the calibration data.

We changed the force range of the texture sensor by using springs with different spring constants. Therefore, four types of springs, 5.9, 24.52, 42.85, and 85.81 N/mm, were used to construct the texture sensor, which were termed S6, S25, S43, and S86, respectively. Assuming that each spring was compressed by 3 mm in S6, S25, and S43, and 2 mm in S86, the force ranges of the texture sensor of S6, S25, S43, and S86 from each spring constant were estimated to be 17.7, 73.56, 128.55, and 171.62 N, respectively.

**Figure 3:**
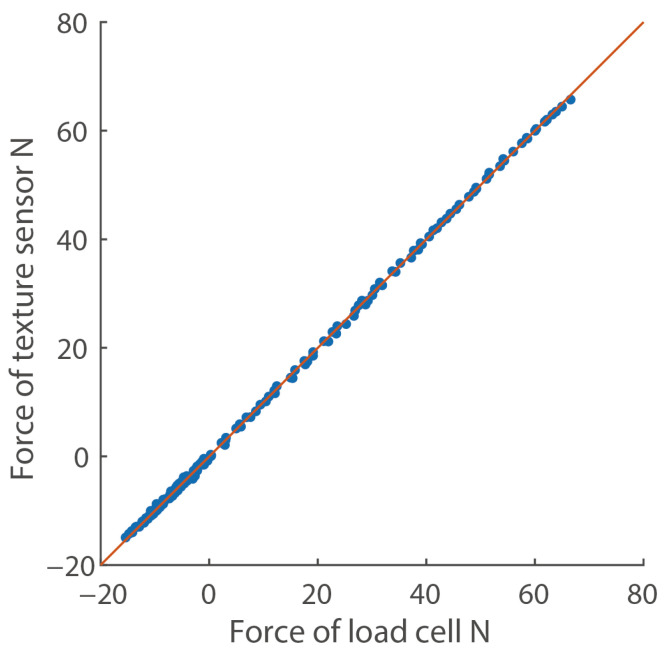
Typical calibration result (n=160). The positive and negative forces indicate the force during pushing and pulling, respectively. The red line shows the approximation line.

Figure 3 shows a typical calibration result of the texture sensor using S25. In the calibration process, the measurement system obtained the data set of the reference force by the load cell and the output voltages by the texture sensor during the 2.5 mm pushing and 0.6 mm pulling and determined the coefficients of Equation (1). The root mean squared error was 0.41 N and the determination coefficient $R^{2}$ was 0.999. By using Equation (1), the calibration with a low error was possible.

### 2.2. Measurement System

**Figure 4:**
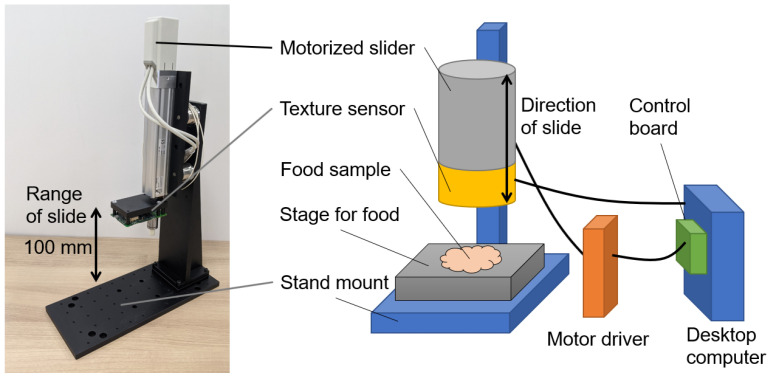
Measurement system for the texture sensor.

A measurement system is shown in Figure 4 and primarily consists of a texture sensor, motorized slider, stand mount, motor driver, control board, and desktop computer. The motorized slider (LEY16DA, SMC Co., Tokyo, Japan) is fixed to the stand mount and connected to the desktop computer via the motor driver (LECPAN1, SMC Co., Tokyo, Japan) and control board (SMC-4DL-PE, Contec Co., Osaka, Japan). The operation of the motorized slider is controlled by commands from the computer. The bottom-up texture sensor attaches to the electric slider and the texture sensor then compresses or breaks up the food in accordance with the slider’s movement.

### 2.3. Distance between the Measured Data Points

The measurement data of the force and vibration by the texture sensor are time series data. In order to quantify the difference between the measurement data and the data to be compared in the experiment, the dynamic time warping (DTW) distance was used [24]. The DTW enables temporal expansion and contraction and determines the correspondence between data elements, minimizing the distance between the time series data. As the data measured by the texture sensor varies over time depending on the individual samples, the DTW distance is more suitable than the Euclidean distance for evaluating differences between the data. In the experiment, the sample was compressed twice. In this case, the force data was the waveform of two peaks, and the DTW could then calculate the appropriate DTW distance by associating the tops of these with each other. On the other hand, as the vibration data is a waveform consisting of many pulses, the DTW could not be applied as it was. Therefore, the DTW was applied to the data obtained by converting multiple pulses into a waveform with several peaks through the sum of the movements. In order to calculate the DTW distance from each set of measurement data, the data to be compared was required. In the experiment, the texture sensor analyzed seven kinds of chicken nuggets, and the average data from each kind was taken as the data to be compared. DTW barycenter averaging (DBA) is an algorithm that determines the average of multiple time series data points [25]. The average data profile for each type of chicken nugget was determined by means of the DBA. The DTW distance between the measurement data and the average indicates the degree of dissimilarity between them. Hence, the DTW distance can be used to ensure that the texture sensor measures the same type of sample as similar waveforms.

### 2.4. Preparation of the Chicken Nuggets

We prepared seven types of chicken nuggets with different coatings to be used as the food samples. The chicken nuggets have the same meat paste inside. Since we can design the mix of the coating and the texture sensor measures mainly the difference among the coatings, we used chicken nuggets. Hereinafter, the seven kinds are referred to as C1 through C7. The main ingredient was ground chicken leg meat. A food processor rendered meat paste from the leg meat, and a mixed seasoning was then applied. All of the nuggets were composed of the same meat paste. The paste was placed in molds to create a uniformly cylinder nugget shape and then frozen at −40 °C. A mold-shaped nugget had a size of 47 × 12 mm (in diameter and thickness) and weighed 20 g. Seven different mixtures of a batter coating developed with flour were then coated onto the mold-shaped nuggets. Three of the seven nugget samples were coated with breader breadcrumbs. Table 1 presents the materials of the batter coating and breaders. After being coated, the nugget samples were frozen at −40 °C. Table 2 compares texture sensations of seven chicken nuggets. We prepared 20 samples for one kind of chicken nugget. In an experiment, all of the samples were fried in vegetable oil at 180 °C for 3 min; their force and vibration were then measured after a waiting period of 6 min at the room temperature. Table 3 presents the mean and standard deviations of the coating thicknesses. Since these coatings have different physical parameters, the texture sensor measures the difference as measurement data.

## 3. Experiments and Results

### 3.1. Fundamental Evaluation of the Magnetic Food Texture Sensor

#### 3.1.1. Range

**Figure 5:**
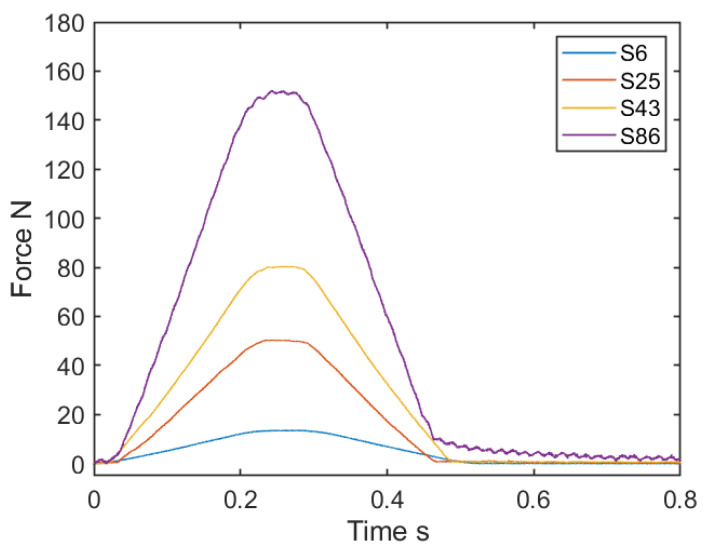
Force measured based on the 2 mm displacement of the probe of the four texture sensors equipped with different springs.

We performed experiments in order to check the texture sensor’s force range. In one experiment, the motorized slider pushed the probe against an acrylic plate suitably placed on the measurement system. As the distance pushed from the contact state of the probe was 2 mm, this corresponds to a displacement of the probe by 2 mm. After this displacement, the motorized slider moved the texture sensor upwards until it was separated from the plate. The texture sensor then measured the force during the motorized slider’s motion. Figure 5 shows the force measured by the texture sensors, from S6 to S86. The vertical axis represents the force calculated using the calibration formula, whereas the horizontal axis represents the time. It took about 0.5 s from the start of pushing until the probe separated from the plate. The maximum forces of each texture sensor were reached at about 0.25 s, at which point the forces were about twice the spring constant. After separating from the plate, S86 gradually approached 0 N, which indicates that it takes time for the spring to return to its natural length. Therefore, this result reveals that the response characteristics of S86 are not good. On the other hand, the other three sensors returned to 0 N at 0.5 s.

#### 3.1.2. Resolution

**Figure 6:**
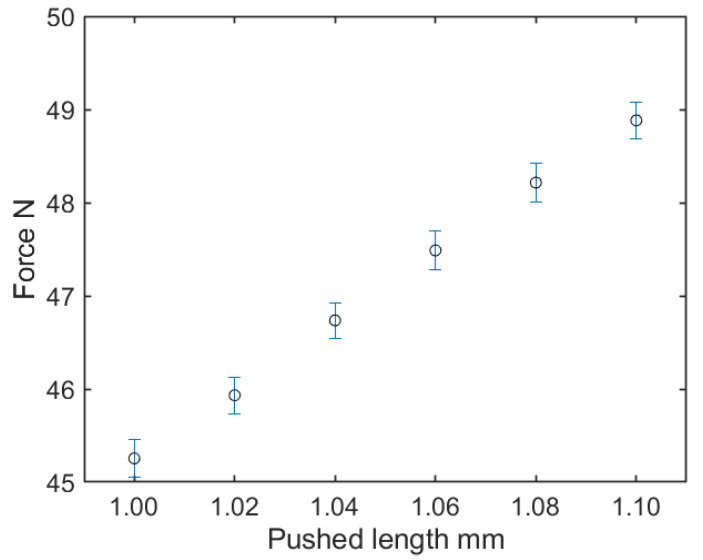
Relationship between the pushed length and the force of S43.

As in the previous section, the texture sensor’s probe was pushed against the acrylic plate by the motorized slider. Initially, the texture sensor was pushed 1 mm from its contact state with the plate and force measured for 1 s. Then, additional pushes of 0.02 mm were made and measured for 1 s a further five times. As a typical result, the relationship between the pushed length of S43 and the force is displayed in Figure 6. Based on the measured force, the mean and standard deviation of the force at each pushed length, and the mean of the differences in force caused by the 0.02 mm displacement of the probe, were calculated. The results are summarized in Table 4. According to Figure 6, the force linearly changed with the 0.02 mm displacement. Moreover, the error bars of adjacent forces did not overlap, and this tendency was the same for other sensors with different spring constants. With respect to the mean of the force differences induced by the 0.02 mm displacement of the probe shown in Table 4, it was observed that the larger the spring constant, the larger the mean. However, the means of force difference of each texture sensor were smaller than twice their standard deviations. This indicated that the resolution of the texture sensor was smaller than the force difference caused by the 0.02 mm displacement of the probe, which was less than 1% of the force range assumed in Section 2.1.3.

#### 3.1.3. Durability for Repetition

**Figure 7:**
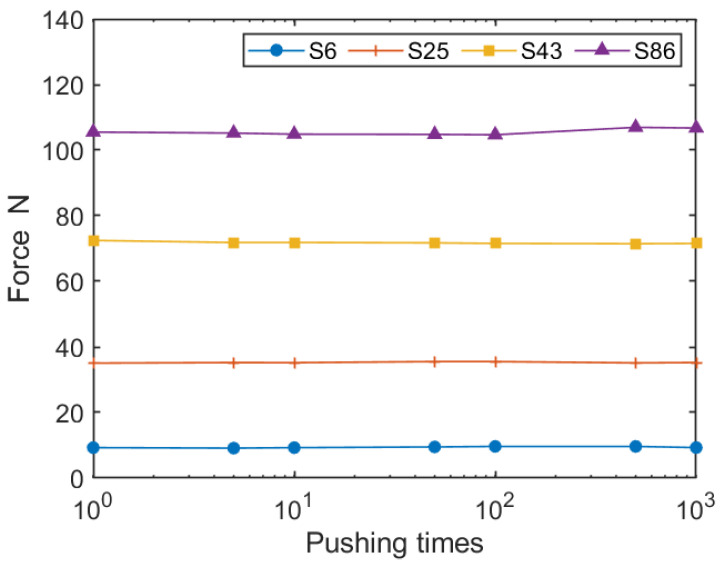
Representative force measured out of 1000 times by the four texture sensors.

Each texture sensor from S6 to S86 was pushed against the plate by the motorized slider, displacing the probe by about 1.5 mm, after which the force was measured; then, the motorized slider lifted the texture sensor by 1.5 mm to return it to the contact state. This process of operation and measurement was repeated 1000 times. Figure 7 shows the forces measured the 1st, 5th, 10th, 50th, 100th, 500th, and 1000th times. The horizontal axis of the graph is logarithmic. According to Figure 7, it was confirmed that the force did not change in a particular direction over 1000 repetitions and remained almost constant.

#### 3.1.4. Differences in the Frequency Response

**Figure 8:**
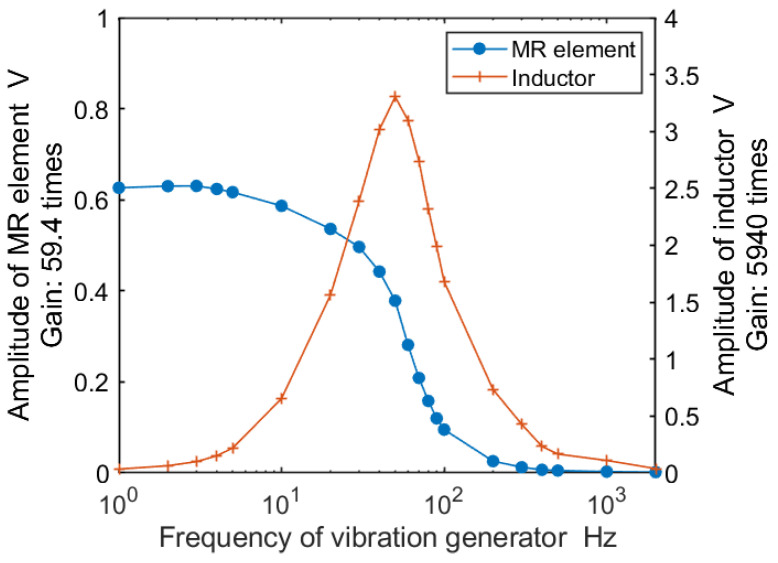
Relationship between the frequency and amplitude in terms of the voltage of the MR element and inductor in the vibration range from 1–2000 Hz.

In order to confirm the differences in response between the MR element and inductor, a vibration generator periodically displaced the probe of the texture sensor. Vibrations were made in the 1–2000 Hz frequency. Figure 8 shows the relationship between the frequency and amplitude in terms of the voltage of the MR element and inductor of the texture sensor with S43. As the vibration generator had a constant ampere for generating the vibrations, the amplitude of the vibration length decreased. This decrease in vibration amplitude is reflected in the decrease in the amplitude of the MR element, which began to decrease from 10 Hz and was almost 0 at 200 Hz. On the other hand, the amplitude of the inductor gradually increased from 10 Hz, reaching a maximum value of 3.5 V at 50 Hz; it was about 0.17 V at 500 Hz and 0.11 V at 1000 Hz. As the noise of the inductor voltage, when there was no displacement in the probe, was about 0.02 V, the result means that even a vibration of 1000 Hz could be measured. Comparing the MR element and inductor, the frequency responses differed and the MR element sensitively responded to frequencies of 100 Hz or less and the inductor to frequencies of 10 Hz or more and 1000 Hz or less.

### 3.2. Analysis of Measurement Data of Chicken Nuggets

#### 3.2.1. Measurements

**Figure 9:**
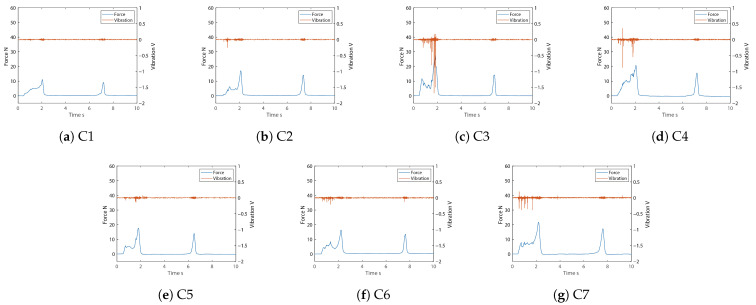
Typical force and vibration data of seven nugget samples.

The system used to measure the nugget samples operated using two-time compressions. The degree of compression was 90%. The number of samples of each type of chicken nugget was 20, and a total of 140 measurements were conducted. The typical force and vibration data of the seven nugget samples are shown in Figure 9. With respect to the force, the first peak indicates the response to the first compression and expresses the difference of the coating. The second peaks in Figure 9 show almost the same height because the coatings have broken by the first compression and the second one mainly compressed the meat. If a coating of the sample had many small fractures from the compression, the waveform of the first peak was repeated over small ups and downs, e.g., C3, C4, C6, and C7. The higher first peak indicates the harder coating, e.g., of C3, C4, and C7. With respect to the vibration, the number of voltage spikes refers to the number of vibrations, and their heights represent the intensities of the vibrations. The C3, C4, and C7 samples featured many vibrations. In particular, the vibration of C3 was large, which indicates that large fractures occurred. The measurement data of each sample show the differences in the respective textures of the nugget coatings.

#### 3.2.2. Difference in the Measurement Data

**Figure 10:**
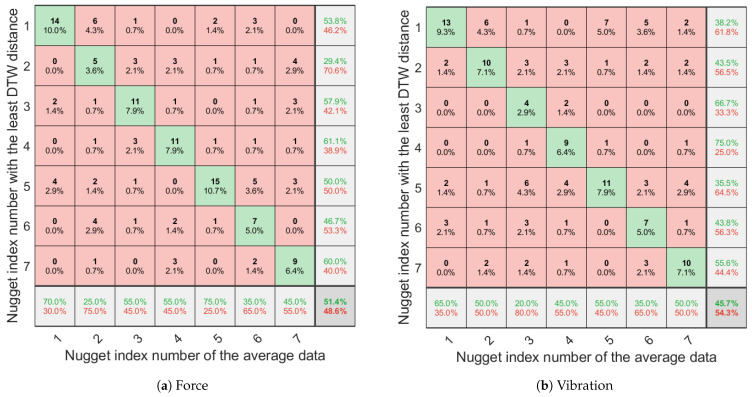
Confusion matrixes of force and vibration. The numbers and percentages were calculated for when the DTW distance in the average data for nuggets with the same coating was least compared to the DTW distances for the average data of other nuggets with different coatings. The green number indicates the percentage which the nugget index number with the least DTW distance and the nugget index number of the average data matched. The red numbers indicate the percentages that did not match.

First, the average data for each kind of nugget were calculated on the basis of the measurement data. Second, the DTW distances between the seven average data and all measurement data were determined by a round-robin protocol. It was expected that the DTW distance between the average data and measurement data of nuggets with the same coating would be lesser. Third, for some of the measurement data, the percentages were calculated for when the DTW distance in the average data for nuggets with the same coating was least compared to the DTW distances for the average data of other nuggets with different coatings. Confusion matrixes of force and vibration are shown in Figure 10. $F_{1}$ scores of each nugget sample are also presented in Table 5. For force, all nuggets except C2 and C6 had $F_{1}$ scores above 0.5, and the mean of the percentage in the lower-right corner of Figure 10a exceeded 50%. For vibration, only C4 and C7 had $F_{1}$ scores higher than 0.5, while the others had $F_{1}$ scores lower than 0.5. The mean of the percentage in the lower-left corner of Figure 10b was also lower than 50%. The average data of force captured the features of each coating better than those of vibration.

## 4. Discussion

In this study, we developed a magnetic food texture sensor to simultaneously measure force and vibration occuring by means of a probe. Using the prototype texture sensor, we conducted a series of fundamental experiments to confirm the range, resolution, repetitive durability of force, and frequency response. As Figure 5 shows, we confirmed the force range of 10, 50, 80, and 150 N using four types of springs. These ranges correspond to, for instance, sliced apples, raw green beans, raw pears, and raw carrots [1]. We can employ the texture sensor’s full range by appropriately replacing the spring on the basis of the object’s hardness. The spring of the texture sensor is composed of plated iron and affects the magnetic field generated by the permanent magnet it contains. As the texture sensor was calibrated to include the effect of the spring on the magnetic field, it is considered that stable force measurement was even possible with the magnetic spring.

In the experiment conducted for the resolution, we confirmed the force and its error by means of repetitive pushes of 0.02 mm. The resolutions of the four texture sensors were less than 1% of the force range, and the error was less than the resolution. The force was calculated from the voltages of the two MR elements using Equation (1). As the noise in the voltage was small, the texture sensor had a small resolution. Even if the texture sensor is equipped with a spring with a high spring constant, the common AD conversion ports in the circuit board ensure that the resolution is almost constant, as the AD conversion ports in the circuit board are common. This means that we can estimate the resolution of the force when replacing the spring.

With respect to repetitive durability, as Figure 7 displays, the force was almost constant, with virtually no tendency to modulate up and down over 1000 repetitions of pushes. This indicates that each sensor had sufficient durability for at least 1000 uses. However, 1000 tests are not enough for industrial applications, and for this about 10,000 tests will be required. As the guaranteed number of compressions of the spring is 300,000, the texture sensor could continue the measurement for 10,000 repetitions, but the durability of the other components is not guaranteed. For instance, the probe was produced by a 3D printer, and it may break after 1000 repetitions. Furthermore, the shape of the texture sensor’s probe generally depends on the object of measurement. The probe used in this study is cylindrical in shape, but wedge-shaped or wide and flat surfaces could be required. Further confirmation of durability is therefore needed, including probes with shapes other than cylindrical.

The texture sensor features two different elements. As shown in Figure 8, the MR element responds with high sensitivity to frequencies of 100 Hz or less, whereas the inductor responds to frequencies of 10–1000 Hz. As the instruments used in TPA mainly feature load cells for measuring force, they are limited to measurements in the low-frequency band, such as the MR element in this study. On the other hand, the texture sensor described herein measures the displacement and vibration generated in a probe with different elements. Some researchers have suggested the presence of rapidly adapting mechanoreceptors in the periodontal ligament [20,26]. The range of their response frequencies has not been reported, but when referring to the response frequencies of rapidly adapting mechanoreceptors in the skin, Bolanowski et al. reported that the high-frequency range for the perception of vibration was from 40 to 500 Hz [27]. The inductor of the texture sensor satisfied this range. The frequency response of the inductor was considered suitable for measuring textures with sudden fractures, such as arising from crispness and crunchiness qualities. In order to evaluate fracturing textures, the combination of the MR element and the inductor will be important.

To confirm the effectiveness of the texture sensor for the measurement of food, seven kinds of chicken nuggets with different coatings were evaluated. As is shown in Figure 9, there was some difference in the waveforms of force and vibration, but only on the basis of differences in the coatings. Although the TPA only determines the physical properties from the force data, it is considered difficult to evaluate textures because each sample has individual differences. In this study, the average data on force and vibration for each type of nugget was determined by means of DBA, and the DTW distance between the average data and measured data was then calculated. As Figure 10 indicates, the DTW distance between the average data and measured data of the same kind was least at the highest percentage of 75% and the lowest of 20%. The mean percentages were 51.4% and 45.7% for the force and vibration, respectively, which means that about half of the samples could be discriminated when the DTW distance was used as an evaluation value. In Table 5, the force $F_{1}$ scores of C1, C3, and C5 were higher than the vibration, and vice versa for C2. The others differed below 10 in terms of $F_{1}$ score of force and vibration. This result indicates that C1, C3, and C5 are better characterized by force data than vibration, and vice versa for C2. In other words, the texture sensor that simultaneously measures force and vibration captured different features of the textures of the coatings. In this experiment, the evaluation was performed on the basis of the DTW distance, as the distance between the two sets of data was calculated after expanding and contracting in the time direction and aligning the peaks. As this is a rough comparison though, we would like to analyze and compare the area of force and the numbers and heights of the vibration spikes in greater detail in future research.

The main limitations of this study are as follows. The durability over 1000 repetitions was investigated, but durability beyond this number and in accordance with aging could not be evaluated. In order to quantify the features of the force and vibration, we employed the DTW distance. Moreover, other features should be used, e.g., physical quantities and the geometrical features of the waveform. The experiment dealt only with chicken nuggets with different coatings. The experimental results also depend on only the cylindrical probe. However, it would be necessary to further evaluate the texture sensor for a wide range of targets that also generate vibration, such as potato chips and crackers.

## 5. Conclusions

In this study, we developed a magnetic texture sensor equipped with an industrial spring, a linear slider, and magnetic components. The range, resolution, repeatability of force, and difference in the frequency response of the MR element and inductor were evaluated via a set of experiments, and we outlined the texture sensor’s basic characteristics. Moreover, the texture sensor measured seven kinds of chicken nuggets with different coatings, and we confirmed that it was effective for capturing the features of the different coatings on the basis of force and vibration data. In future research, we will develop a method for texture evaluation based on both force and vibration data.

## Figures and Tables

**Table 1:** Materials of the batter and breader mixes. The unit is g.

**Materials of the Batter Mix**	**C1**	**C2**	**C3**	**C4**	**C5**	**C6**	**C7**
Wheat flour	70	80	40	20	80	80	10
Acetylated oxidized starch	30	10	25	0	20	20	0
Acid-treated starch	0	0	0	20	0	0	0
Oxidized starch	0	0	0	30	0	0	30
Pregelatinized cornstarch	0	0	0	0	0	0	20
Distarch phosphate	0	0	35	30	0	0	40
Dextrin	0	10	0	0	0	0	0
Paprika pigment	0.4	0.4	0.4	0.4	0.4	0.4	1
Leavening agent	0	0	0	0	0.3	0.3	0
Xanthan gum	0	0	0	0	0	0	0.1
Cold water	120	120	100	100	140	140	140
**Materials of the Breader Mix**	**C1**	**C2**	**C3**	**C4**	**C5**	**C6**	**C7**
Wheat flour	90	60	-	-	-	0	-
Pregelatinized cornstarch	0	20	-	-	-	100	-
Distarch phosphate	0	20	-	-	-	0	-
Fat powder	10	0	-	-	-	0	-
Leavening agent	0.2	0	-	-	-	0	-

**Table 2:** Texture sensations of seven chicken nuggets. Compared among nuggets, they were roughly evaluated when blending materials of coating.

Index of Nuggets	Texture Sensations
C1	Less both crispness and crunchiness in samples
C2	Repetitive crunchiness with a relatively weak force
C3	Continuous crispness with a relatively strong force
C4	Repetitive crunchiness with a relatively strong force
C5	Short crispness with a relatively weak force
C6	Continuous crispness with a relatively weak force
C7	Short crispness with a relatively strong force

**Table 3:** Mean and standard deviations of the thickness of the fried nuggets’ coatings. Different letters (*a*–*c*) indicate significant differences (p<0.05) among the samples (n=20).

	C1	C2	C3	C4	C5	C6	C7
Thickness mm	2.8 ± $0.5{\;}^{a}$	$3.2\pm 0.5{\;}^{b}$	$2.1\pm 0.3{\;}^{c}$	$2.6\pm 0.5{\;}^{a}$	$1.9\pm 0.4{\;}^{c}$	$3.3\pm 0.4{\;}^{b}$	$2.4\pm 0.5{\;}^{ac}$

**Table 4:** Mean and standard deviations of the force at each pushed length and mean of the difference in force caused by the probe’s 0.02 mm displacement. Each force had significant differences (p<0.05) among forces in the same row (n=10).

Sensor	Mean and Standard Deviation of Force in 1 s Intervals						Mean of Difference
	1.00 mm	1.02 mm	1.04 mm	1.06 mm	1.08 mm	1.10 mm	
S6	7.41 ± 0.05	7.56 ± 0.05	7.71 ± 0.05	7.84 ± 0.05	7.96 ± 0.05	8.17 ± 0.05	0.15
S25	29.17 ± 0.19	29.61 ± 0.19	30.30 ± 0.19	30.80 ± 0.18	31.57 ± 0.18	32.12 ± 0.18	0.59
S43	45.26 ± 0.20	45.93 ± 0.20	46.74 ± 0.19	47.49 ± 0.20	48.21 ± 0.20	48.88 ± 0.20	0.72
S86	79.54 ± 0.31	80.25 ± 0.32	81.60 ± 0.31	82.63 ± 0.30	83.94 ± 0.31	85.54 ± 0.32	1.20

**Table 5:** $F_{1}$ scores of each nugget sample in Figure 10.

	C1	C2	C3	C4	C5	C6	C7
Force	0.61	0.27	0.52	0.59	0.60	0.40	0.51
Vibration	0.48	0.47	0.31	0.56	0.43	0.39	0.53

## Data Availability

The data presented in this study are available on request from the corresponding author.

## Abbreviations

The following abbreviations are used in this manuscript:

DTW	Dynamic Time Warping
DBA	DTW Barycenter Averaging

## References

[1] Bourne M.C. (2002). Texture, Viscosity, and Food.

[2] Abbott J.A. (1999). Quality measurement of fruits and vegetables. Postharvest Biol. Technol..

[3] Nishinari K., Fang Y. (2018). Perception and measurement of food texture: Solid foods. J. Texture Stud..

[4] Franceschelli L., Berardinelli A., Dabbou S., Ragni L., Tartagni M. (2021). Sensing Technology for Fish Freshness and Safety: A Review. Sensors.

[5] Szczesniak A.S. (2002). Texture is a sensory property. Food Qual. Prefer..

[6] Szczesniak A.S. (1963). Objective Measurements of Food Texture. J. Food Sci..

[7] Szczesniak A.S. (1963). Classification of Textural Characteristics. J. Food Sci..

[8] Friedman H.H., Whitney J.E., Szczesniak A.S. (1963). The Texturometer—A New Instrument for Objective Texture Measurement. J. Food Sci..

[9] Pellegrino R., Cheon B.K., Forde C.G., Oleszkiewicz A., Pieniak M., Luckett C.R. (2020). The contribution of texture contrasts and combinations to food acceptance across cultures. J. Texture Stud..

[10] Saeleaw M., Schleining G. (2011). A review: Crispness in dry foods and quality measurements based on acoustic-mechanical destructive techniques. J. Food Eng..

[11] Chen J., Karlsson C., Povey M. (2005). Assessment of Biscuits. J. Texture Stud..

[12] Varela P., Chen J., Fiszman S., Povey M.J. (2006). Crispness assessment of roasted almonds by an integrated approach to texture description: Texture, acoustics, sensory and structure. J. Chemom..

[13] Taniwaki M., Hanada T., Sakurai N. (2006). Device for acoustic measurement of food texture using a piezoelectric sensor. Food Res. Int..

[14] Taniwaki M., Kohyama K. (2012). Mechanical and acoustic evaluation of potato chip crispness using a versatile texture analyzer. J. Food Eng..

[15] Salvador A., Varela P., Sanz T., Fiszman S.M. (2009). Understanding potato chips crispy texture by simultaneous fracture and acoustic measurements, and sensory analysis. LWT Food Sci. Technol..

[16] Akimoto H., Sakurai N., Shirai D. (2017). A new device for acoustic measurement of food texture using free running probe. J. Food Eng..

[17] Sakurai N., Akimoto H., Takashima T. (2021). Measurement of vertical and horizontal vibrations of a probe for acoustic evaluation of food texture. J. Texture Stud..

[18] Kato S., Wada N., Ito R., Shiozaki T., Nishiyama Y., Kagawa T. (2019). Snack texture estimation system using a simple equipment and neural network model. Future Internet.

[19] Nakamoto H., Nishikubo D., Kobayashi F. (2018). Food texture evaluation using logistic regression model and magnetic food texture sensor. J. Food Eng..

[20] Piancino M.G., Isola G., Cannavale R., Cutroneo G., Vermiglio G., Bracco P., Anastasi G.P. (2017). From periodontal mechanoreceptors to chewing motor control: A systematic review. Arch. Oral Biol..

[21] Goka M., Nakamoto H., Takenawa S. A magnetic type tactile sensor by GMR elements and inductors. Proceedings of the 2010 IEEE/RSJ International Conference on Intelligent Robots and Systems.

[22] Liu Y., Han H., Liu T., Yi J., Li Q., Inoue Y. (2016). A Novel Tactile Sensor with Electromagnetic Induction and Its Application on Stick-Slip Interaction Detection. Sensors.

[23] Kawasetsu T., Horii T., Ishihara H., Asada M. (2018). Mexican-Hat-Like Response in a Flexible Tactile Sensor Using a Magnetorheological Elastomer. Sensors.

[24] Sakoe H., Chiba S. (1978). Dynamic programming algorithm optimization for spoken word recognition. IEEE Trans. Acoust. Speech Signal Process..

[25] Petitjean F., Ketterlin A., Gançarski P. (2011). A global averaging method for dynamic time warping, with applications to clustering. Pattern Recognit..

[26] Maeda T., Ochi K., Nakakura-Ohshima K., Youn S.H., Wakisaka S. (1999). The Ruffini ending as the primary mechanoreceptor in the periodontal ligament: Its morphology. Crit. Rev. Oral Biol. Med..

[27] Bolanowski S.J., Gescheider G.A., Verrillo R.T., Checkosky C.M. (1988). Four channels mediate the mechanical aspects of touch. J. Acoust. Soc. Am..

